# Scarf Injury: a qualitative examination of the emergency response and acute care pathway from a unique mechanism of road traffic injury in Bangladesh

**DOI:** 10.1186/s12873-022-00698-2

**Published:** 2022-08-04

**Authors:** Anna Tupetz, Eleanor Strand, Kazi Imdadul Hoque, Mohsina Sultana, Joao Ricardo Nickenig Vissoci, Catherine Staton, Michel D. Landry

**Affiliations:** 1 Duke Global Health Institute, Duke University, Durham, NC USA; 2 Department of Emergency Medicine, Duke University Medical Center, Durham, NC USA; 3 International Committee of the Red Cross (ICRC), Savar, Bangladesh; 4 Center for the Rehabilitation of the Paralyzed (CRP), Savar, Bangladesh; 5 Directorate of General Health Services (DGHS), Government of Mohakhali, Dhaka, Bangladesh; 6 Western Norway University of Applied Sciences, Bergen, Norway; 7 Virginia Commonwealth University, Richmond, VA USA

**Keywords:** Spinal Cord Injury, Traumatic Spinal Cord Injury, Scarf Injury, Bangladesh, Emergency Care, Acute Care, Trauma, Road Traffic Injury, Road Traffic Accident, Isadora Duncan Syndrome, Long-Scarf Syndrome, Strangulation

## Abstract

**Background:**

Road traffic injuries (RTI) are the leading cause of death worldwide in children over 5 and adults aged 18–29. Nonfatal RTIs result in 20–50 million annual injuries. In Bangladesh, a new mechanism of RTI has emerged over the past decade known as a ‘scarf injury.’ Scarf injuries occur when scarves, part of traditional female dress, are caught in the driveshaft of an autorickshaw. The mechanism of injury results in novel, strangulation-like cervical spine trauma. This study aimed to understand the immediate emergency response, acute care pathway, and subsequent functional and health outcomes for survivors of scarf injuries.

**Methods:**

Key informant interviews were conducted with female scarf injury survivors (*n* = 12), caregivers (*n* = 6), and health care workers (*n* = 15). Themes and subthemes were identified via inductive content analysis, then applied to the three-delay model to examine specific breakdowns in pre-hospital care and provide a basis for future interventions.

**Findings:**

Over half of the scarf injury patients were between the ages of 10 and 15. All but two were tetraplegic. Participants emphasized less than optimal patient outcomes were due to unawareness of scarf injuries and spinal cord injuries among the general public and health professionals; unsafe and inefficient bystander first aid and transportation; and high cost of acute health care.

**Conclusions:**

Females in Bangladesh are at significant risk of sustaining serious and life-threatening trauma through scarf injuries in autorickshaws, further worsened through inadequate care along the trauma care pathway. Interventions designed to increase awareness and knowledge of basic SCI care at the community and provider level would likely improve health and functional outcomes.

**Supplementary Information:**

The online version contains supplementary material available at 10.1186/s12873-022-00698-2.

## Background

Road traffic injuries (RTI) are the leading cause of death worldwide in children over 5 and adults aged 18–29 [1]. Beyond mortality, RTIs result in 20–50 million annual injuries [2]. Spinal cord injuries (SCI) are among the most severe [2]. SCI survivors navigate a high degree of lifelong disability and a two- to five-fold increase in the chance of premature death [3].

Surviving a traumatic SCI, and subsequent functional outcome is dependent on the quality of emergency care within the first 72 h of the RTI [4]. Clinical practice guidelines recommend immediate spinal stabilization and rapid, safe transport to a trauma-equipped health facility [5]. In low- and middle-income countries (LMIC), however, most of the population lacks access to a formal pre-hospital care system and ambulance transport [6]. An estimated 93% of all fatal RTIs occur in LMICs [1]. For individuals that survive, inadequate post-crash care results in poor functional outcomes [2].

In Bangladesh, an LMIC in southeast Asia, a new mechanism of RTI has emerged over the past decade known as a ‘scarf injury.’ A scarf injury occurs when a scarf, part of the traditional female dress (i.e., ‘upa’ or ‘orna’), becomes entangled in the drive shaft of the Easy Bike, an autorickshaw-type taxi. While males can wear a scarf around their neck, especially during colder temperatures, these scarves are worn differently and from a thicker material, and thus do not expose males to the same risk as females thereby highlighting gender-based differences in Bangladesh. The scarf wraps around the neck, strangles the woman, and forcefully pulls her to the ground off the vehicle. Strangulation results in severe laceration and a cervical SCI, often a complete lesion of the spinal cord.

The literature, most in the form of case reports, describes the mechanism of scarf injury in Ghana, India, and Pakistan [7–15]. To our knowledge, no studies have examined pre-hospital care. This study, therefore, aimed to understand the immediate emergency response, acute care pathway, and subsequent functional and health outcomes for scarf injury survivors through the lived experience of patients, caregivers, and health care workers (HCW). The three-delays model, a framework originally designed for maternal mortality, was applied to examine specific breakdowns in pre-hospital care and provide a basis for future intervention [16].

## Methods

### Study design

In late 2017, clinician-researchers from the Centre for the Rehabilitation of the Paralyzed (CRP) in Savar, Bangladesh approached the senior author (MDL) about a series of SCI patients presenting with a new mechanism of injury now known as a scarf injury. CRP requested to collaborate and conduct a study on scarf injuries across the continuum of care in order to better understand the mechanism of injury, influencing factors to receiving adequate care, and subsequent long-term health and quality of life. This manuscript focuses on the emergency response and acute care pathway of scarf injury patients until admission to CRP.

A mixed-methods approach centered on key informant interviews with scarf injury patients, caregivers, and health care workers was selected. The study design was further informed by experts and consultants in qualitative research and research ethics at Duke University.

Data collection was conducted between June and July 2018. Eligible patient participants were selected from an overall registry of SCI patients at CRP. The registry included a subset of individuals who presented to CRP with scarf injuries between 2013 and 2018 and served as the initial list of possible study participants. Prior to departure from Bangladesh, the head researcher briefed CRP on preliminary results and main challenges identified by study participants to access care.

### Setting

CRP has 12 locations across Bangladesh and provides medical and rehabilitation services, primarily for neurological disorders [17]. The study was conducted at the main center in Savar. To our knowledge, CRP-Savar was the only medical facility in Bangladesh with in-patient care and rehabilitation services specifically for SCI patients.

### Research team and reflexivity

The research team in Bangladesh consisted of a female researcher and physical therapist (AT) and two rehabilitation professionals based at CRP active in the treatment of SCI patients (MS and KS). AT, with previous experience in qualitative research methods served as the head researcher. KS was the first to discover scarf injuries at CRP and has intensively worked with every scarf injury patient admitted to CRP from 2013 to 2018. AT, alongside a female translator, conducted home visits (patients) and interviews with patients, caregivers, and health care workers.

The research team in the United States (US) consisted of an emergency medicine physician (CS) with vast previous experience in global health research, qualitative injury research, and motivational interviewing; a psychologist (JRNV) with expertise in global health research and qualitative analysis; an undergraduate student with prior experience in qualitative data analysis and gender-based inequities (ES); and a senior rehabilitation and disability researcher (MDL) with over 15 years of experience, specifically in Bangladesh.

Scarf injury patients were not involved in the study design, but female SCI survivors from CRP were actively involved in interview guide development and content validation of emerging themes. This decision was made by US and Bangladesh research team members.

### Participant selection

Patient participants were identified based on purposive sampling strategies. A registry of patients admitted to CRP was used to create a list of patients who fit the study criteria. Eligibility criteria required all of the following were met: 1) female, 2) former CRP patient, 3) treated for a scarf injury caused by riding passenger on an Easy Bike between 2014 and 2018, 4) diagnosed with a complete or incomplete cervical SCI, 5) lives with at least one caregiver, 6) discharged from CRP at least three months prior to the interview, 7) were a minimum of ten years old at the time of scarf injury, and 8) able to communicate in Bengali/Bangla or English. The resulting registry list included 40 patients. 10 were deceased at the time of recruitment. A total of 30 scarf injury patients were thus identified. The registry data served as an additional source of demographic patient information to complement the qualitative data.

Caregiver participants were selected as an extension of patient participants in order to better understand the experiences surrounding the injury and clinical care, as well as the impact the injury had on the whole family unit. The caregivers were able to complement the patient interviews with additional information by lending a different perspective on the challenges along the care continuum.

HCW participants were selected via purposive sampling. A list of all disciplines involved in the rehabilitation of scarf injury patients at CRP was compiled by MS and KH.

### Recruitment

**Fig. 1:**
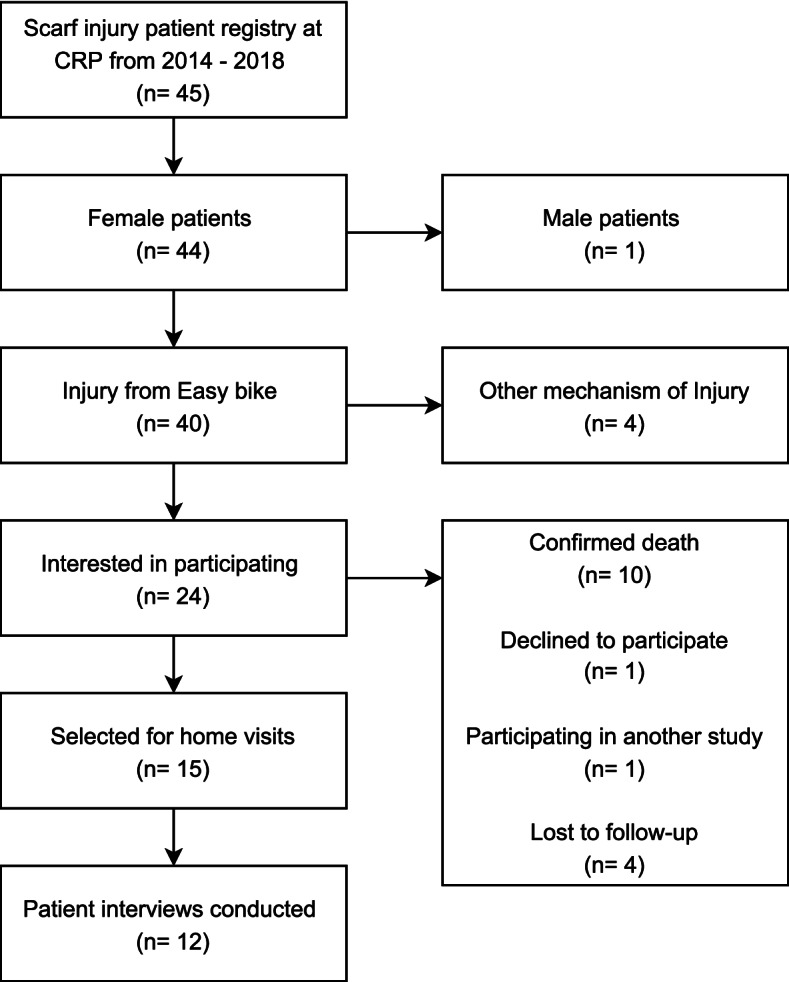
Flowchart of scarf injury patient participant recruitment

The study was introduced over the phone to all 30 scarf injury patients. During recruitment, four were lost to follow up, one was participating in an unrelated study, and one declined to participate. In the case of patients under age 18, the team first received consent from the legal guardian before speaking with the patient to receive assent. After confirmation of interest and consent, the possible study population was 24 scarf injury patients. A maximum variation sample was attempted by selecting patients to participate based on age at the time of scarf injury, time since scarf injury, level and severity of scarf injury, and geographic location of residence and/or injury. Maximum variation was thwarted, to a small degree, by logistical challenges to reach participant homes. Therefore, patients were organized and invited for interviews based on logistics and maximum variation. Data collection terminated when information saturation was reached. As described in Fig. 1, a total of 12 scarf injury patients were interviewed. Caregiver participants were invited to participate and complete a key-informant interview during the home visits to conduct patient interviews. For the HCW participants, the manager and/or supervisor of each discipline, identified by MS and KH, were approached and asked to share a study invitation to participate with their team. Interested participants contacted AT to schedule an interview. Recruitment ended when maximum variation across disciples was reached.

### Interview procedure

In July 2018, interviews were conducted in the patient participant homes across Bangladesh. Home visits were chosen to obtain an impression of living conditions and functional independence. Each home visit lasted two to four hours. The average interview length was one hour, including back and forth translations.

Patient participants had the option to decide whether caregivers and family members were present during the interviews. During the interviews, the researcher asked the questions in English, which were then synchronously translated into Bengali, the participants’ local language. Participant responses were translated from Bengali to English. The translator was a healthcare professional and graduate student fluent in Bengali and English and not known to the study participants. The translator was trained by MS, KH, and AT on the interview guide and conducted pilot interviews. The audio recordings of the first round of participant interviews were then reviewed by KH and discussed with the translator to provide feedback on the translation process. The recording of the first patient interview was evaluated and discussed with the research team. Based on this discussion, we performed another training session with the translator and slightly adjusted the interview guide structure (Appendix 1).

The interview guide was developed, aiming to understand the experiences and perceptions of patients and caregivers, following the biopsychosocial model and quality of life domains. The guide was initially developed by the head researcher and revised in several feedback rounds with qualitative experts, as well as researchers and clinicians in Bangladesh, to ensure cultural appropriateness and relevance of questions.

All interviews were digitally audio-recorded, and the audio recordings were transcribed. To assess information saturation, we discussed field notes after each interview and identified the main topics and information covered in each interview. After 10 home visits, the research team determined no new topics were emerging. After an additional two interviews, we confirmed saturation was reached, and our sample was a good representation of the population.

The HCW participants were interviewed at CRP by AT. The participants had the option to conduct the interview in English or through the help of a translator. The HCW interview guide was developed in collaboration between MS, KH, AT and MDL, and focused on better understanding the clinical needs, challenges, and available resources of scarf injury patients (Appendix 2).

### Data analysis

The final qualitative methodology was rooted in a phenomenological approach [18]. Data was analyzed through inductive content analysis [19] and applied to the three-delay model [16]. An independent translator translated the participants’ responses from Bangla to English and transcribed the English versions. All complete English transcripts were then proofread by an independent but experienced researcher fluent in Bangla and English.

For the coding process, using QRS International NVivo12, we first split each interview broadly into themes based on the interview guide [20]. Emergent themes from the interviews were then coded by the head researcher who conducted the interviews, and the codebook was created.

We created the codebook through a lumping and splitting process, revising, and discussing with peer researchers throughout the analysis process. To ensure coding rigor, a subset of interviews was double coded to compare the coding strategies and ensure coding consistency. Differences in coding were discussed until agreement was reached. After the coding, analytic memos on parent codes were written and shared with the research team.

We discussed apparent emergent themes with the research team in Bangladesh to get their cultural input on interpreting the preliminary findings. After the conclusion of formal data analysis, four cases (three patients and one mother) were presented as vignettes to two females at CRP who have been paraplegic for over ten years. One works as a peer supporter and has extensive experience with the challenges young female scarf injury patients experience. The other is a member of the wheelchair basketball team that practices at CRP. The four cases were chosen by AT as representative of the breadth of responses and all main emergent themes. Case presentations included a summary of the interview findings as well as an interpretation based on the analysis. A local clinician researcher discussed these cases with the two females and received their feedback on the accuracy of the interpretations.

### Ethics statement

Ethical Approval was obtained from Duke Health Institutional Review Board, Protocol ID: Pro00092024, as well as CRP Ethics Committee, Bangladesh, Protocol ID: CRP-R&E-0401–224. Depending on the level of literacy, written or verbal informed consent was obtained from the study participants. Verbal consent was documented and signed by the interviewer in the presence of the study participant. The translator served as a witness during the consent procedure and also signed the consent form. If the participant was under the age of 18, the participant provided assent and the legal guardian provided consent.

## Results

### Demographics and characteristics of study participants

In-depth interviews were conducted with scarf injury survivors (*n* = 12) (Table 1), caregivers (*n *= 6), and health care workers (*n* = 15). All caregiver interviewees were mothers. Half were present at the time of injury and immediate emergency response, and all were involved in the subsequent acute care pathway.

**Table 1:** Scarf injury interview participants and Scarf injury registry demographics

	Participants (*n* = 12)	Full Patient Registry (*n* = 40)
Age at time of injury (years)		
10–15	4 (33.3%)	17 (42.5%)
16–20	4 (33.3%)	6 (15%)
21–35	4 (33.3%)	17 (42.5%)
Characteristics		
Occupation (pre-injury)		
Student	8 (66.6%)	25 (62.5%)
Housewife	3 (25%)	11 (27.5%)
Other	1 (8.3%)	4 (10%)
Married (pre-injury)	4 (33.3%)	17 (42.5%)
Injury Details		
Complete lesion	10 (83.3%)	28 (70%)
Surgery performed	5 (41.6%)	18 (45%)
Ligature mark	12 (100%)	36 (90%)
Secondary complications upon arrival to CRP		
Pressure ulcer	1 (8.3%)	9 (22.5%)
Respiratory complaints	5 (41.6%)	25 (62.5%)
Time from injury to CRP (days)*	48 (1; 215)	61 (1; 413)
Time since injury (months)*	30 (9; 54)	34 (9; 56)

Note: *CRP* Center for the Rehabilitation for the Paralyzed

^*^Range: min–max

Health care workers (HCW) were health professionals involved in the treatment of scarf injury patients at CRP, the rehabilitation center. Professions included medical doctors, nurses, physiotherapists, occupational therapists, speech language pathologists, and counselors working in different roles across the care continuum and clinical care provision of scarf injury patients.

**Fig. 2:**
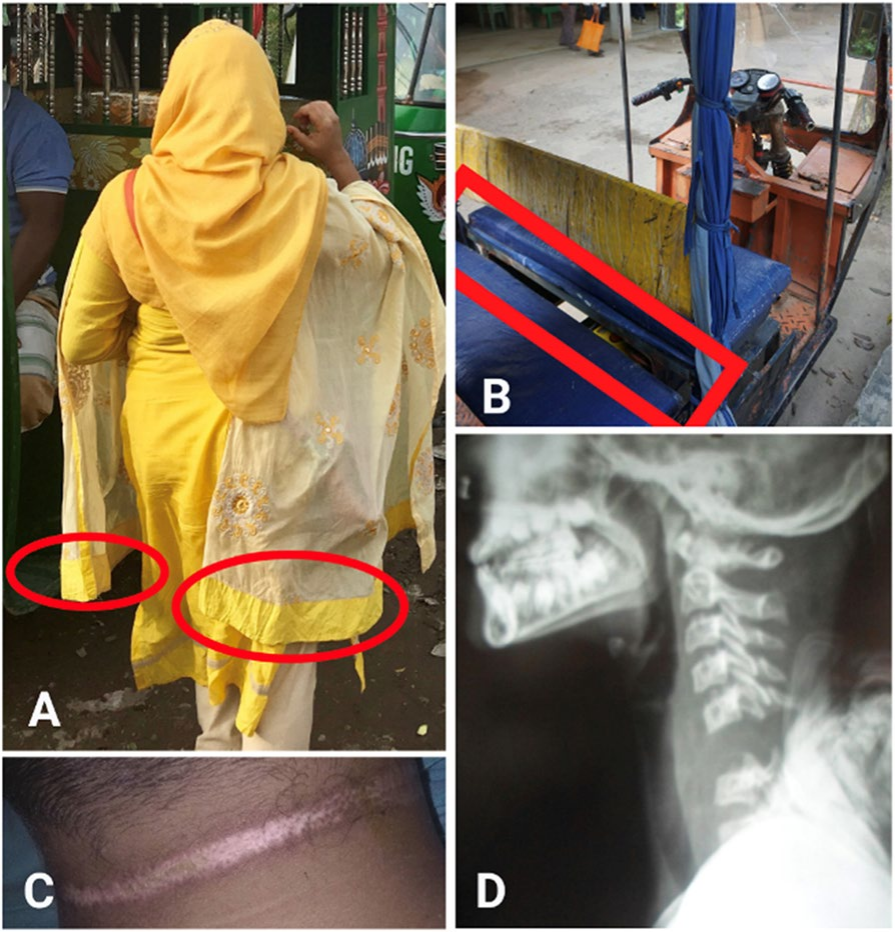
Mechanism of scarf injury and clinical presentation. (**A**) Visual presentation of the traditional scarf worn by a female. The red circles visualize the ends of the scarf that can slide into the gap in the easy bike and cause strangulation (**B**) Visualization of the Easy Bike. A red box delineates the gap between the passenger and driver seats where the scarf is caught and pulled into the driveshaft. (**C**) Ligature mark, present in 90% of scarf injury patients, from the forceful pull. (**D**) X-ray of the head and neck. A case with complete lesion (i.e., severing of the spinal cord) is pictured

The average age of scarf injury patients was 18, with half (*n* = 6) between the ages of 10 and 15. All survivors were passengers on the Easy Bike when the scarf injury occurred. The level of injury (LOI) was cervical and ranged from C3 to C6. Just under half of patients (*n* = 5) had an LOI at C4. Almost all scarf injuries resulted in complete lesions of the spinal cord (*n* = 10), meaning patients had limited to no sensory or motor function below the LOI and were tetraplegic. The patients with incomplete lesions (*n* = 2) were able to regain near pre-SCI function, reporting no limitations in movement other than occasional pain in the neck and/or shoulder (Fig. 2).

Given the Easy Bike was deemed to be the cheapest form of transport by our study participants, two HCWs reported low-income women are at highest risk for scarf injury and compose the majority of the scarf injury population. This phenomenon is not reflected in the CRP patient registry data. These HCWs theorized the registry patients are the scarf injury survivors who were able to afford acute care treatment and the cost of rehabilitation at CRP. It is not known what happens to survivors that cannot afford treatment.

Almost all HCWs reported a significant difference between the general SCI population and the scarf injury population. Scarf injuries were argued to be the most severe due to 1) almost all patients are female (gender roles and inequities in Bangladesh), 2) most patients are under the age of 18, and 3) the mechanism of injury almost always results in tetraplegia.

“In [these] children, it is not like cancer. They will have to live for a long time. And if the family do not have money, then they have nothing left. They do not have any place in society. And also [no place] in school. They do not get that much of support.” — HCW 12.

### Emergent themes and the three-delay model

Inductive content analysis of the patient, caregiver, and HCW in-depth interviews revealed five distinct themes (Table 2**).** Theme 1 describes the scarf injury incident and mechanism of injury. Themes 2, 3, and 4 reflect the three-delays framework for mapping access to adequate health care [16]. Theme 5 surrounds participant recommendations for improving the emergency response and acute care pathway.

**Table 2:** Emergent themes and subthemes of patient, caregiver, and HCW interviews

Themes	Subthemes
Description of injury	Mechanism of injury Incidence Comparison to other SCIs Mortality
Delay in decision to seek care	Awareness of scarf injury/SCIs First aid response
Delay in reaching care	Awareness of scarf injury/SCIs First aid response Transportation
Delay in receiving adequate care	Awareness of scarf injury/SCIs Acute care until CRP Secondary complications Cost of care
Participant recommendations	Advice of patients and caregivers Acute care recommendations

#### Description of injury (Theme 1)

All patients reported sitting behind the driver on the Easy Bike when the injury occurred. Most were one of multiple passengers and on the way to or from community activities with family and/or friends. The women and girls wore scarves either with one end in the front and one end in the back or with both ends in the back.

Patients, caregivers, and HCWs described the mechanism of injury (MOI) in the same way. The scarf slipped into the gap between the passenger and driver seat, became entangled in the drive shaft, and strangled the woman or girl. Patients reported feeling an instant and forceful pull. The majority fell off the Easy Bike to the ground and a few lost consciousness. The force of strangulation resulted in ligature marks in all patients.

Multiple HCWs emphasized the MOI made scarf injuries more severe than other traumatic SCIs. The LOI would often be higher due to the position of the scarf on the neck, and the sheer force exerted by the motor results in severe respiratory difficulties.

The incidence of scarf injuries was not well understood. Based on CRP patient registry data, annual incidence was around 10, but over half the HCWs theorized it to be much higher. Two HCWs estimated 1 in 10 scarf injury survivors would make it to CRP; one HCW estimated 1 in 20.

“Scarf injury, in general, is a very horrible event. A lot of people [are] unaware about these things. A lot of patients die on the spot. And maybe we do not know about them. And a lot of patients are admitted in different hospitals, tertiary level hospitals, different districts. And you don't know about that.” — HCW 8.

In terms of mortality, the majority of HCWs stressed that most scarf injuries result in “spot death,” a local expression referring to death at the scene of injury. A HCW hypothesized “spot death” occurred when the LOI was at C1 or C2. If patients were not “spot dead,” multiple HCWs reported most women and girls would die within the upcoming months.

#### Delay in decision to seek care (Theme 2)

10 of 12 patient-caregiver pairs reported not knowing about scarf injuries and spinal cord injuries prior to their injury. All HCWs emphasized unawareness among patients, caregivers, and the general public.

“I realized that there is certain disease like this. I never had known that hands and legs become paralyzed if something wrong happens on neck. I didn’t know that.” — Patient 4.

90% of patients and caregivers reported that family, friends, and/or bystanders at the scene conducted the immediate emergency response. One caregiver-patient pair received help from a firefighter. Most patients were unable to communicate to those trying to help due to strangulation and/or loss of consciousness.

A few patients were brought home after the scarf injury. When their condition did not improve, family and/or friends sought traditional and/or modern health care.

#### Delay in reaching care (Theme 3)

No patients were transported from the injury site to a health facility in an ambulance. All were transported in an Easy Bike or another rickshaw-type vehicle. This required moving the patient. When reflecting on the scarf injury incident, most patients and caregivers hypothesized movement exacerbated the injury.

“They didn’t understand that [she] became paralyzed, so they made her sit. But, she didn't have balance, [and] again she fell down on the middle of the vehicle facing downwards. The damage occurred more that time.” — Caregiver 12.

Multiple HCWs referred to this as “mishandling.” One HCW estimated 10–15% of scarf injuries started out as incomplete lesions and became complete through mishandling in the emergency response (including transportation) and subsequent acute care pathway. HCWs, patients, and caregivers noted rough road conditions, in addition to mishandling, contributed to further injury.

“When injury occurred at the road, the general population in our country, they don't know about the general aid. They don't know about the first aid. Regarding injury, they just pull the body and [take] another vehicle to the hospital. We call it poor handling. And the cervical region [is] getting more and more injury.” — HCW 9.

After the immediate emergency response, most patients were transported to modern health facilities. A few were brought to traditional healers. Multiple HCWs reported that traditional care worsened scarf injuries and resulted in poorer functional outcomes (i.e., mishandling). After not improving, patients who sought traditional care then went to modern health facilities.

“In rural [regions], there is poor education [and] falls from high trees [are] very common. In our religion, [these are] superstitions. When someone falls from height, they [think] maybe it did something with his or her [spirit]. And they take her and treat her with traditional medicine. And that makes the injury much worse because lack of spinal positioning.” — HCW 8.

All patients and caregivers reported being denied care at the first modern health facility. Denial of care was due to health professionals either recognizing the facility was unable to treat the injury or perceiving the injury as a suicide attempt.

“They took me to [primary] health complex. The doctor of the clinic [was] frightened after seeing my condition and didn’t touch me. He referred me to [different primary] health complex. He said, ‘We can’t manage this patient. Take her [to different primary] health complex.” — Patient 3.

A HCW explained that, in Bangladeshi culture, suicide was highly stigmatized and considered a criminal offense. This HCW confirmed the ligature mark from the scarf looks similar to that from hanging and could be misdiagnosed as an attempted suicidal hanging.

#### Delay in receiving adequate care (Theme 4)

After initial denial of care, patients went to a minimum of 2, maximum of 6, additional health facilities before the SCI was diagnosed and treated. In most cases, patients were transported via ambulance to subsequent health facilities and received the actual SCI diagnosis and treatment in Dhaka, the capital of Bangladesh.

Patients and caregivers described the care received prior to Dhaka as inadequate. In all cases, the health professionals did not recognize the scarf injury as an SCI. The severity of injury was underestimated and treatment, if given, focused treatment on the neck laceration (i.e., ligature mark).

“[The] doctor made her stand. [The] doctor wasn’t understanding anything. My sister was screaming. [I was saying] like, ‘Brother, it’s not a simple problem, her scarf [got] entangled on her neck, her neck sprained, [her] face turned behind!’ Still, our village doctors weren’t understanding. They forcefully made her stand [and] she couldn’t stand, her knees were bending. Then, after seeing her, he gave [her] medicines for three days. The doctor said she will be fine within three days.” — Caregiver 6.

Some patients and caregivers described financial cost as a barrier to continued acute care due to the high number of health facilities. The highest amount reported on healthcare expenditures was 5 lakh (500 000 Bangladeshi Taka (approximately 5500 USD) covering costs such as surgery medical supplies (traction device), hospital admissions, ambulance transports.

“It took 3 lakhs taka for surgery in those 15 days at [name redacted] hospital.

Then bed sore developed and we went there again, stayed 19 days and this time it cost 1 lakh money. […] Some money came from [my] daughter’s salary. My son mortgaged some land. We had trees [and] cows. We sold those. Allah helped us. We were continuing expenditure somehow by grace of Allah.” — Caregiver 2.

Once the SCI was diagnosed in Dhaka, appropriate treatment—surgical care and/or spinal traction—was delivered. Patients and caregivers, however, reported issues with spinal traction, including incorrect use and breaking of the apparatuses. HCWs echoed these concerns, emphasizing most health professionals outside of CRP are not trained in proper treatment and management of SCIs. One HCW noted the standard nursing curriculum in Bangladesh did not teach SCI patient management. In addition to lack of awareness regarding scarf injuries, all patients, and caregivers, as well as most health professionals outside CRP, were not aware of CRP as a specialized facility to care for SCIs.

“We didn't even know about CRP. If we would [have] known about CRP earlier, then we would [have] go to CRP first. In Dhaka, we visited so many clinics, but no one would admit her.” — Caregiver 12.

All HCWs reported that unawareness of scarf injuries, SCIs, and CRP among patients, caregivers, and non-CRP health professionals resulted in poorer functional and health outcomes. The majority of patients were admitted to CRP with secondary complications. 1 in 4 scarf injury patients arrived with pressure sores and 2 in 3 arrived with respiratory complaints. HCWs, patients, and caregivers reported urinary tract infections (UTI) as another common secondary complication. Multiple HCWs described cases in which secondary complications delayed rehabilitation and limited functional potential.

#### Participant recommendations (Theme 5)

To prevent scarf injuries and/or improve patient outcomes, all HCWs and patients recommended there be a general awareness campaign on scarf injuries and SCI first aid (e.g., positioning).

“First, we should make an awareness program. Any place it can happen, we can give a board-like structure. Wherever it is happening, [we teach to] place the body on the board, so the alignment will be the same level. [If not], mishandling will happen, [and] more disability after injury. […] So, the scarf injury incident [itself] can be preventable, and after the injury, more injury can also be preventable if they know these things.”—HCW 4.

In addition, multiple HCWs recommended a wide-scale education and/or training program on basic SCI treatment and patient management for all health facilities and professionals.

## Discussion

**Fig. 3:**
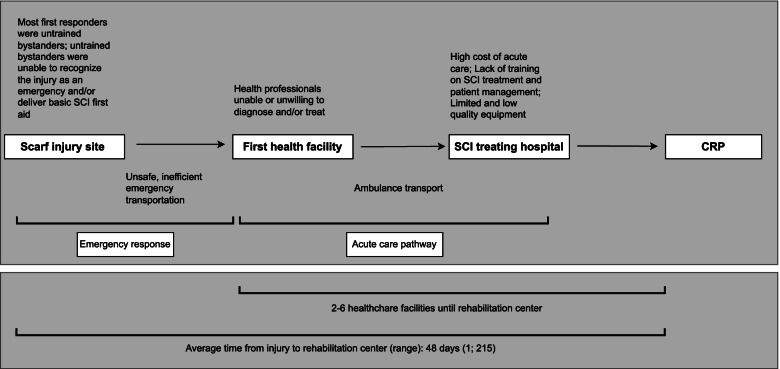
Challenges identified within the immediate emergency response and acute care pathway. Participants emphasized less than optimal patient outcomes were due to unawareness of scarf injuries and spinal cord injuries among the general public and health professionals; unsafe and inefficient bystander first aid and transportation; and high cost of acute health care. (SCI = Spinal Cord Injury; CRP: Center for the Rehabilitation of the Paralyzed) *maximum reported costs spent on receiving medical care: 500 000 Bangladeshi Taka (approx. 5500 USD)

This study explored the emergency response and acute care pathway for scarf injury survivors through the lived experience of patients, caregivers, and HCWs. Organizing results within the three-delay model revealed the significance of and gaps within the post-injury care continuum (Fig. 3). The three-delay model conceptualizes access to health care through categorizing barriers into three distinct delays: 1) delay in decision to seek care, 2) delay in reaching care, and 3) delay in receiving adequate care [16].

Health and functional outcomes of traumatic SCIs are highly dependent on access to adequate care within 72 h of injury [4]. Based on patient-caregiver narratives and HCW testimonies, all three delays resulted in less-than-optimal patient outcomes. Delays in reaching care, and associated mishandling, were of particular emphasis. In accordance with the literature, the lack of an effective emergency response and safe, available transportation had the largest negative impact on patient outcomes [21].

A recent study in Dhaka, Bangladesh concluded poor access to emergency transport was a result of decentralized ambulance services and lack of national oversight [22]. These challenges extend to several other LMICs [21]. In Kenya, an LMIC in East Africa, a mobile app known as LifeFlare was developed and implemented as a potential solution. LifeFlare links subscribed callers to a national network of private ambulance providers. Thus far, LifeFlare has augmented prehospital care in Kenya and might be an option for Bangladesh [23].

Patients, caregivers, and HCWs also emphasized lack of awareness of scarf injuries and SCIs across all three delays. The first delay is rooted in lack of awareness among the public, including patients, caregivers, and bystanders. This unawareness manifested within the second delay as mishandling. The well-intended, but detrimental, efforts of bystanders is a critical finding, in particular for LMICs with underdeveloped prehospital care systems. The prehospital care system in Bangladesh offers a national emergency number, but access is limited based on region, training is not provided to and certifications are not required of prehospital providers, and no national level system evaluations have been conducted [24].

In 2013, a large-scale study on the emergency response across rural Bangladesh found that of 115,000 non-fatal injuries, 82% of victims received first aid. Of those, 3% received first aid from a trained person or provider [25]. The emergency response to scarf injuries aligns with these data; all but one patient received first aid and transportation from bystanders. These bystanders were unaware of scarf injuries, SCIs, and basic SCI first aid (e.g., do not bend the spine). According to the WHO, “even the most sophisticated and well-equipped prehospital trauma care systems can do little if bystanders fail to recognize the seriousness of a situation, call for help, and provide basic care until help arrives” [6].

HCWs recommended education and/or awareness campaigns as potential solutions. Further, the growing success of task-shifting efforts in low-resource, high-demand settings suggests training lay people or taxi drivers as first responders could be a solution [21]. To reach a large audience, approaches such as wide scaled education video dissemination through social media formats offer an opportunity to scale access to key information. Until the past decade or so, however, little attention was given to emergency care in LMICs and limited literature exists regarding models for improving prehospital systems on a systemic and national scale [26].

In terms of health professionals outside CRP, lack of knowledge regarding scarf injuries and the proper treatment and management of SCIs manifested as denial of care in the second delay and as the third delay itself. Lack of appropriate training foci has also been identified as a barrier to effective emergency care in other LMICs [26]. In Bangladesh, culture, in particular the stigmatization of suicide, factored into denial of care. Training efforts, for health professionals and for the lay public, must be context- and culture-specific in order to maximize impact [27].

Cost of treatment was an additional barrier in continuing acute care. Several participants reported healthcare expenditures exceeding or even doubling the amount of the Gross National Income per capita in Bangladesh. Difficulties in affording health care is common across LMICs, and it is not unusual for families to fall into debt [28]. Bribery to access care, especially in public health facilities, is prevalent across Bangladesh and a further challenge to receiving and continuing emergency care [28, 29].

Lastly, and perhaps most significantly, the fact scarf injuries almost exclusively impact females cannot (nor should not) be ignored. Historically, injury prevention and treatment efforts have focused on the group most at risk: males. Across the world, males are more likely to suffer an RTI than females and three times as likely as to die [1]. Women and girls tend to experience nonfatal RTIs. Trauma among females, however, is underreported [30] and scarf injuries, most of which are fatal, contradict these patterns. Current RTI interventions, in Bangladesh and other LMICs, remain focused on males and do not protect or support female RTI patients [31–33]. Further, women and girls are less likely to receive adequate post-injury care than males due to gender inequities [30, 34]. Additional research is needed to quantify and respond to the gender-specific burdens of RTIs across the emergency response and acute care pathway in LMICs.

## Limitations

The patient and caregiver sample was representative of the whole CRP patient registry, but limited in its small size and inherent selection bias. These patients were the most fortunate of the true scarf injury population given all received enough emergency and acute care to survive and had enough resources to make it to CRP. The magnitude of challenges across the emergency response and acute care pathway were likely underestimated. In terms of HCWs, the sample was representative of the workforce at CRP, but did not include health professionals along the prehospital and acute care continuum. Health professionals outside CRP would have provided better estimates of “spot death” and incidence and allowed direct insight into the level of knowledge regarding scarf injuries and SCI treatment and patient management. Due to logistic challenges, it was not possible to receive patient and caregiver feedback on the results and overall findings. However, a small group of females living with SCIs and a peer support worker reviewed and validated the findings within the cultural context.

## Conclusion

Females in Bangladesh are at significant risk of sustaining serious and life-threatening trauma through scarf injuries. This study applied the three-delay model to map challenges across the emergency response and acute care pathway and identify potential intervention points. Interventions designed to increase awareness and knowledge of basic SCI care at the community and provider level would likely improve health and functional outcomes.

## Supplementary Information

**Additional file 1.**

## Data Availability

Given the rarity of the injury within at the data collection site, it is not possible to fully de-identify the identity of the participants and as such the datasets and/or analyzed during the current study are not publicly available. Redacted codebooks are available from the corresponding author on reasonable requests.

## Abbreviations

SCI: Spinal cord injury
LMIC: Low- and middle-income country
RTI: Road traffic injury
HCW: Health care worker
CRP: Centre for the Rehabilitation of the Paralyzed
LOI: Level of injury
MOI: Mechanism of injury
