# Supplementary material for: Scarf Injury: a qualitative examination of the emergency response and acute care pathway from a unique mechanism of road traffic injury in Bangladesh
Source: BMC Emerg Med. 2022 Aug 4;22:141. doi: 10.1186/s12873-022-00698-2 (PMC9351164; doi:10.1186/s12873-022-00698-2)
Supplement: Supplementary file 1 — Additional file 1. [file 12873_2022_698_MOESM1_ESM.docx]

**Appendix**

**Appendix 1. Patient and caregiver interview guide structure**

| **Topic** | **Description** |
| --- | --- |
| Introduction and demographics | Living situation, family members, first insight into current level of activities and preparation |
| Reconstruction of the event and access/delivery of immediate care | Mechanism of injury, clinical outcomes, access to care, experiences of medical care received until discharge |
| Life after the injury and discharge | Daily life, challenges of living with a disability, biopsychosocial model, quality of life domains (WHOQOL, 2012) |
| Identification of needs to regain (disability-adjusted) independence and future goals | Coping mechanisms and influencing factors for community reintegration, advice for other scarf injury patients or caregivers |

**Appendix 2. Health care worker interview guide structure**

| **Topic** | **Description** |
| --- | --- |
| Introduction and demographics | Professional background, experience in treating SI as well as general SCI populations. |
| Knowledge and awareness of SI and perceived awareness of general public | Mechanism of injury, clinical outcomes, access to care, perceived challenges in accessing care, severity of injury |
| Rehabilitation and long-term care | Services provided, rehabilitation care needs, challenges of living with a disability |
| Differences of female SI patients vs general SCI population | Identification of gender and SI specific challenges and needs |
| Support systems, long-term health needs | Available support systems, need for ongoing support, recommendations to improve trauma care pathway (WHO Emergency Care System Framework, 2018) |

**References:**

World Health Organization, WHO Quality of Life WHOQOL, available at: <https://www.who.int/publications/i/item/WHO-HIS-HSI-Rev.2012.03>, last accessed: July 5, 2021

World Health Organization, WHO Emergency care system framework, available at: <https://www.who.int/publications/i/item/who-emergency-care-system-framework> , last accessed: July 8, 2021
